# Genome-Wide Linkage Analysis of Large Multiple Multigenerational Families Identifies Novel Genetic Loci for Coronary Artery Disease

**DOI:** 10.1038/s41598-017-05381-2

**Published:** 2017-07-14

**Authors:** Yang Guo, Fan Wang, Lin Li, Hanxiang Gao, Stephen Arckacki, Isabel Z. Wang, John Barnard, Stephen Ellis, Carlos Hubbard, Eric J. Topol, Qiuyun Chen, Qing K. Wang

**Affiliations:** 1 Center for Cardiovascular Genetics, Department of Molecular Cardiology, Lerner Research Institute, Cleveland Clinic, Cleveland, OH 44195 USA; 2 Department of Molecular Medicine, Cleveland Clinic Lerner College of Medicine of Case Western Reserve University, Cleveland, OH 44195 USA; 3 Heart Center, the First Affiliated Hospital, Lanzhou University, Lanzhou, Gansu 730000 P. R. China; 4 Shaker Heights High School, Shaker Heights, OH 44120 USA; 5 Department of Quantitative Health Sciences, Cleveland Clinic, Cleveland, OH 44195 USA; 6 Department of Cardiovascular Medicine, Sydell & Arnold Miller Family Heart & Vascular Institute, Cleveland Clinic, Cleveland, OH 44195 USA; 7 Scripps Translational Science Institute, Scripps Research Institute, Scripps Clinic, La Jolla, CA 92037 USA; 8 Department of Genetics and Genome Sciences, Case Western Reserve University School of Medicine, Cleveland, OH 44106 USA

## Abstract

Coronary artery disease (CAD) is the leading cause of death, and genetic factors contribute significantly to risk of CAD. This study aims to identify new CAD genetic loci through a large-scale linkage analysis of 24 large and multigenerational families with 433 family members (GeneQuest II). All family members were genotyped with markers spaced by every 10 cM and a model-free nonparametric linkage (NPL-all) analysis was carried out. Two highly significant CAD loci were identified on chromosome 17q21.2 (NPL score of 6.20) and 7p22.2 (NPL score of 5.19). We also identified four loci with significant NPL scores between 4.09 and 4.99 on 2q33.3, 3q29, 5q13.2 and 9q22.33. Similar analyses in individual families confirmed the six significant CAD loci and identified seven new highly significant linkages on 9p24.2, 9q34.2, 12q13.13, 15q26.1, 17q22, 20p12.3, and 22q12.1, and two significant loci on 2q11.2 and 11q14.1. Two loci on 3q29 and 9q22.33 were also successfully replicated in our previous linkage analysis of 428 nuclear families. Moreover, two published risk variants, SNP rs46522 in *UBE2Z* and SNP rs6725887 in *WDR12* by GWAS, were found within the 17q21.2 and 2q33.3 loci. These studies lay a foundation for future identification of causative variants and genes for CAD.

## Introduction

Genetic factors contribute to the risk of developing coronary artery disease (CAD) and its major complication, myocardial infarction (MI), which is the result of the accumulation of atherosclerotic plaques in the walls of the coronary arteries^1–3^. Existing knowledge of genetic components affecting the risk of CAD is largely based on results from genome-wide association studies (GWAS), a systematic, unbiased and powerful approach to identify disease-associated variants using population samples. Although the majority of GWAS have focused on European ancestry populations^4–14^, several GWAS were also reported in African Americans^15^, East Asians^16–21^ and South Asians^9, 22^. Due to newly developed SNP imputation methods^23–25^ based on the HapMap project (https://www.ncbi.nlm.nih.gov/probe/docs/projhapmap/) and the 1000 Genome project (http://www.internationalgenome.org/), meta-GWAS is becoming a more popular strategy for CAD and other complex diseases. The largest meta-GWAS recently analyzed 9.4 million imputed SNPs among >185,000 samples and identified 10 novel CAD loci^14^. To date, there have been 65 independent CAD susceptibility loci reported at a genome-wide significance level (i.e., *P* < 5.0 × 10^−8^). The heritability of CAD has been estimated from 40% to 60% by genetic-epidemiologic studies^26^. However, recent studies strongly indicate that GWAS variants cannot fully explain the heritability of CAD, and all published risk variants explained only 10–20% of heritability^13, 14, 27^.

Genome-wide linkage analysis (GWLA) is another systematic and unbiased approach to identify genetic loci for human complex diseases and to search for evidence of major genetic effects. The first GWLA for CAD, conducted in 2000, involved an analysis of 156 affected sibling pairs and revealed two genetic loci on chromosomes 2q21.1–22 and Xq23–26 that were linked to premature CAD^28^. Since then, over ten GWLAs, including our own studies, have identified additional genetic loci for CAD or MI, including 1p34–36,1q25, 2q14.3, 2q36–37.3, 2q13, 3q13, 5q31, 7p14, 8p22, 13q12–13,14q32.3, 15q26.3, 16p13, and 17p11.2–17q21^28–39^. Recently, we have completed a genome-wide linkage scan in a well characterized U.S GeneQuest cohort with 428 nuclear families and identified six novel CAD loci on chromosomes 3p25.1, 3p29, 9q22.3, 9p34.11, 17p12, and 21q22.3^40^. In contrast to aforementioned GWASs, the number of genetic loci identified by GWLA was much smaller and independent, suggesting that many linkage loci remain to be identified in new CAD or MI families^40^.

Most GWLAs for CAD have been conducted in either single large pedigrees or a large number of nuclear families. Increasing the number of family members within families can improve the power of linkage analysis^36, 40, 41^. In this study, we performed a large scale GWLA in a well-characterized U.S. cohort of 24 large, multigenerational CAD families (mean pedigree size = 18). This cohort, referred to as GeneQuest II, was independent from our previously reported GeneQuest cohort with 428 nuclear families^40^. The most attractive feature of the GeneQuest II cohort is the inclusion of extended family members of affected siblings or trios. To our knowledge, this is the largest linkage analysis of multiple large pedigrees to identify genetic loci for CAD, and significant susceptibility loci were identified.

## Results

### Characteristics of 24 large GeneQuest II families

**Figure 1:**
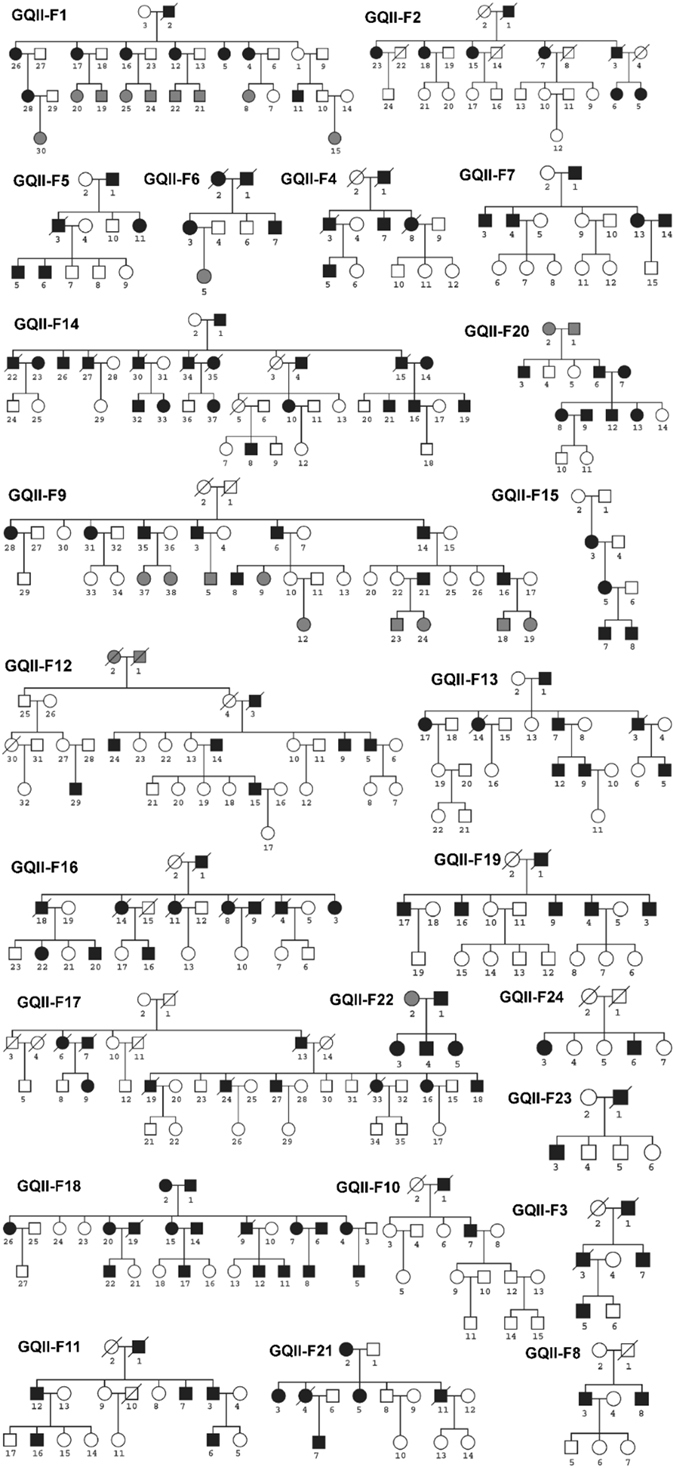
Pedigrees of 24 GeneQust II families. Unaffected subjects were shown as clear circles (females) or squares (males), and affected subjects were shown as solid symbols. Subjects with uncertain phenotypes were shown in gray color. The deceased subjects were marked by a slash symbol.

The 24 large and multigenerational families with CAD and MI were genetically characterized (Table 1). The pedigrees of the 24 GeneQuest II families are shown in Fig. 1. 433 family members from the 24 families were included in the linkage analysis. The pedigree size ranged from 5 to 38 members per family, and the average age of onset of CAD was 51.3 ± 9.2 years in GeneQuest II. There were 162 patients affected with CAD and 247 family members without a diagnosis of CAD. Overall, there were 209 males and 224 females. However, among the CAD group, male patients (107 or 66.0%) were more predominant than female patients (55 or 34.0%). On the contrary, among the non-CAD group, 154 members were females (62.3%). These data are consistent with the notion that the male gender is an important risk factor for CAD.

**Table 1:** Clinical and Demographic Characteristics of the GeneQuest II Study Population.

Feature	GeneQuest II (24 Families)
No. Male/No. Female	209/224
Age of Onset of CAD, year^a^	51.3 ± 9.2
No. affected with CAD	162
Caucasian, %	100
**Pedigree structure**:	
No. of pedigrees	24
Pedigree size, n (mean ± SD)	18.04 ± 10.55
Pedigree size, n (min, median, max)	5, 15, 38
**No**. **of relative pairs**:	
Sibling/sibling, n	398
Sister/sister, n	154
Brother/brother, n	105
Brother/sister, n	139
Half sibling/half sibling, n	0

^a^Data were shown as mean ± SD.

A full set of 410 microsatellite markers spanning the entire human genome by every 10 cM were initially genotyped for all 433 family members in the 24 CAD families. 36 markers were excluded for further analysis, including 9 autosomal markers with genotype and pedigree errors and 27 makers on X and Y chromosomes. Therefore, after quality control, 374 microsatellite markers on autosomes 1–22 from 433 family members were subjected to subsequent statistical analysis.

### Genome-wide linkage scans

As shown in Table 2, genome-wide two-point NPL linkage analysis identified two highly significant linkages at markers D7S3056 (7p22.2, NPL score = 5.19) and D17S1299 (17q21.2, NPL score = 6.20), respectively. Three significant linkages were also identified at markers D2S1384 (2q33.3, NPL score = 4.36), GATA138B05 (5q13.2, NPL score = 4.44) and D9S910 (9q22.33, NPL score = 4.54), respectively (Table 2).

**Table 2:** Genomic Regions Significantly Linked to CAD as Identified by GWLS in the Combined GeneQuest II Families: Two Highly Significant Linkages and Four Significant Linkages.

CAD Locus	Cytoband	Genetic Map^a^	Genomic Position^b^	NPL Score	
				Two-point	Multipoint
**Highly Significant Linkage**: **two**-**point or multipoint NPL** ≥ **4**.**99**					
D17S1299	17q21.2	62.01	38.99	6.20	5.38
D7S3056	7p22.2	7.44	4.49	5.19	4.74
**Significant Linkage**: **two**-**point or multipoint NPL** ≥ **4**.**08**					
D2S1384	2q33.3	200.43	205.23	4.36	4.22
D3S2418	3q29	215.84	192.32	4.00	4.49
GATA138B05	5q13.2	78.80	71.4	4.44	3.35
D9S910	9q22.33	104.48	101.62	4.54	2.73

^a^The genetic map position was based on Marshfield Medical Genetic marker set 11.

^b^Physical genomic position was retrieved from the UCSC database with human build GRCh37/hg19.

**Figure 2:**
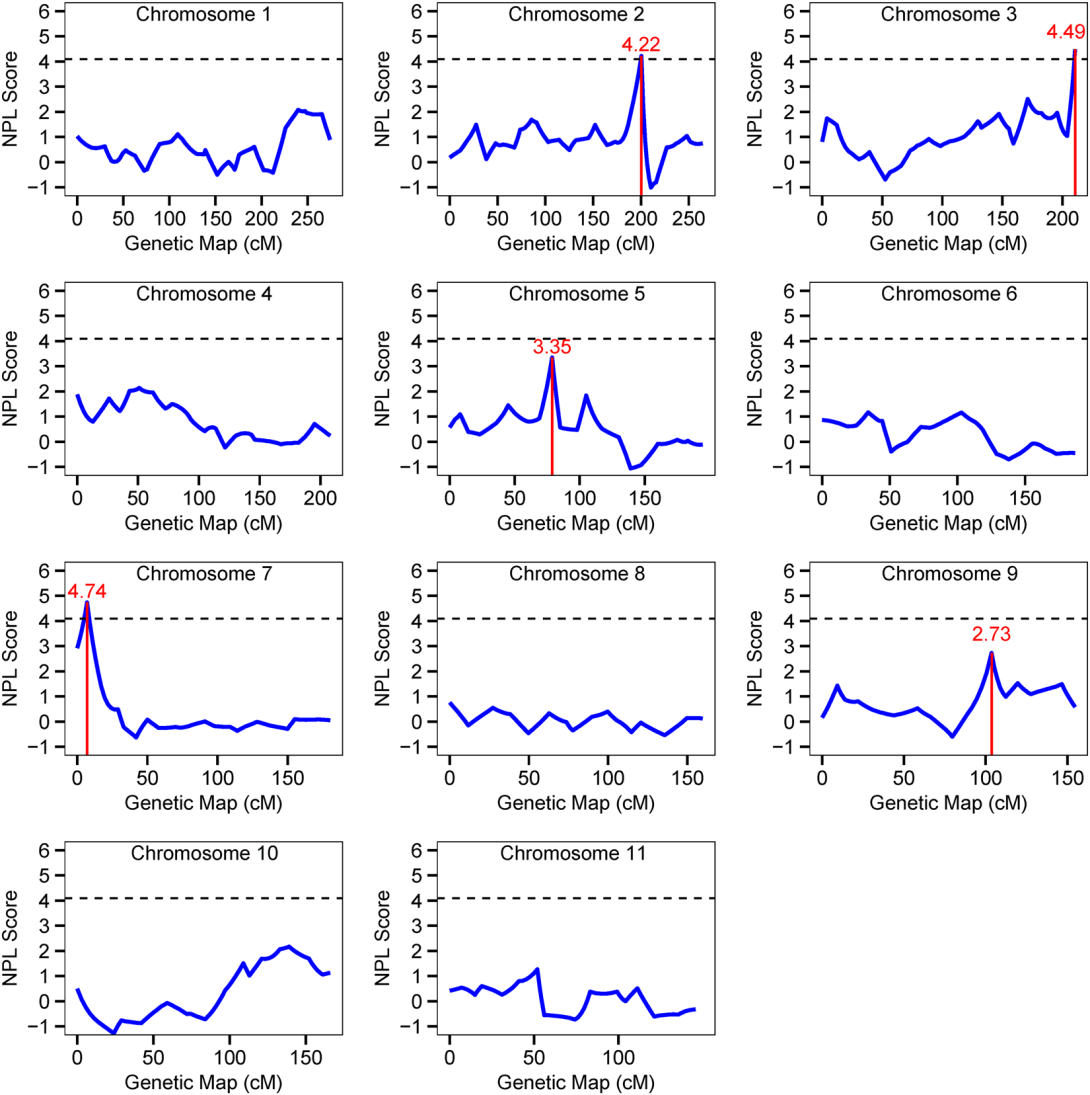
Genome-Wide Linkage Scan of Chromosomes 1–11 for CAD in 24 Large GeneQuest II families. X-axis and Y-axis indicate the genetic map of each chromosome and NPL scores from multipoint linkage analysis as shown by blue solid line, respectively. The red vertical solid line shows a significant linkage peak identified by two-point NPL analysis. The horizontal dash line represents the threshold of significant linkage with a multipoint NPL value of ≥4.08.

**Figure 3:**
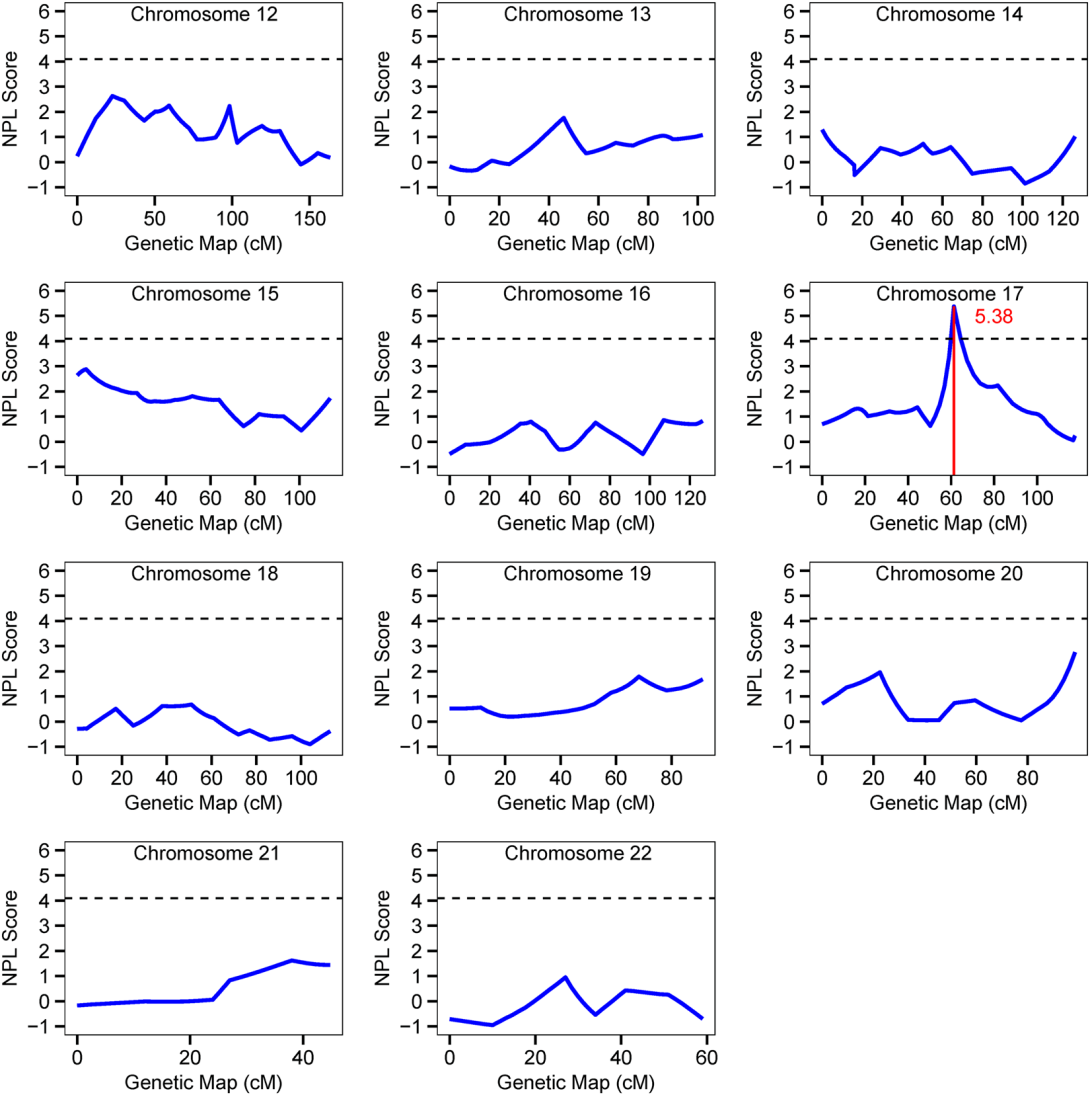
Genome-Wide Linkage Scan of Chromosomes 12–22 for CAD in 24 Large GeneQuest II families. X-axis and Y-axis indicate the genetic map of each chromosome and NPL scores from multipoint linkage analysis as shown by blue solid line, respectively. The red vertical solid line shows a significant linkage peak identified by two-point NPL analysis. The horizontal dash line represents the threshold of significant linkage with a multipoint NPL value of ≥4.08.

Multipoint NPL analysis was further performed. Multipoint NPL scores were plotted along the genetic map for each of 22 chromosomes (Figs 2 and 3). Multipoint NPL analysis identified four significant genetic loci for CAD on chromosomes 17q21.1, 7p22.2, 2q33.3 and 3q29. The top CAD locus on chromosome 17q21.2 identified by the two-point linkage analysis remained to be a highly significant linkage peak with a NPL score of 5.38 by multipoint NPL analysis. The CAD locus on 17q21.2 covered a genetic interval from 56.9 cM to 83.1 cM (Fig. 3). The second best CAD locus identified by multipoint NPL analysis was on 7p22.2 with a NPL score of 4.74, and the linkage covered an interval between 1.4 cM and 11.0 cM (Fig. 2). Compared with two-point NPL scores, multipoint NPL scores of the six CAD loci slightly decreased except for the 3q29 locus with an improved NPL score from 4.00 to 4.49.

Moreover, both two-point and multipoint NPL analyses were carried out in individual families. Each of the 6 significant CAD loci was found to occur in at least one individual family (Table 3). NPL scores in single families were, in general, higher than those in the combined families (Table 3). In addition, this analysis identified 15 new linkages for CAD, including 7 highly significant linkages on chromosomes 12q13.13, 17q22, 20p12.3, 22q12.1, 15q26.1, 9q34.2, and 9p24.2, 2 significant linkages on 11q14.1 and 2q11.2, and 6 suggestive linkages on 10p15.3, 10q21.3, 2p16.3, 20q13.32, 12q23.1, and 4p16.3 (Table 4).

**Table 3:** Six Genetic Loci for CAD Confirmed by GWLS in Individual GeneQuest II Families.

CAD Locus	Cytoband	Family	Genetic Map^a^	Genomic Position^b^	NPL Score	
					Two-point	Multipoint
D17S1299	17q21.2	GQ2-F18	62.01	38.99	16.81	13.22
D17S1299	17q21.2	GQ2-F9	62.01	38.99	3.31	3.95
D7S3056	7p22.2	GQ2-F18	7.44	4.49	12.42	11.27
D7S3056	7p22.2	GQ2-F9	7.44	4.49	3.80	4.47
D7S3056	7p22.2	GQ2-F17	7.44	4.49	3.25	3.43
D2S1384	2q33.3	GQ2-F18	200.43	205.23	16.87	16.83
D3S2418	3q29	GQ2-F14	215.84	192.32	5.66	4.35
GATA138B05	5q13.2	GQ2-F19	78.80	71.40	4.89	3.50
D9S910	9q22.33	GQ2-F18	104.48	101.62	14.41	6.38

^a^The genetic map position was based on Marshfield Medical Genetic marker set 11.

^b^Physical genomic position was retrieved from the UCSC database with human build GRCh37/hg19.

**Table 4:** New Genetic Loci for CAD Identified by GWLS in Individual GeneQuest II Families.

CAD Locus	Cytoband	Family	Genetic Map^a^	Genomic Position^b^	NPL Score	
					Two-point	Multipoint
D12S297	12q13.13	GQ2-F1	65.00	52.61	4.09	6.76
D17S1290	17q22	GQ2-F9	82.00	56.33	2.06	6.51
AAT034	20p12.3	GQ2-F9	25.00	8.71	5.03	6.01
D22S689	22q12.1	GQ2-F9	29.00	28.86	5.92	2.19
D15S652	15q26.1	GQ2-F9	90.00	92.52	3.89	5.83
D9S2157	9q34.2	GQ2-F9	147.00	136.04	5.34	0.87
AAAAC001	9p24.2	GQ2-F9	9.30	4.18	5.00	3.14
D11S2002	11q14.1	GQ2-F18	85.00	79.97	4.83	4.14
D2S2972	2q11.2	GQ2-F1	114.40	102.57	0.61	4.26
D10S1435	10p15.3	GQ2-F1	4.00	2.24	2.79	3.54
D10S1225	10q21.3	GQ2-F14	81.00	64.75	3.51	−0.16
D2S1352	2p16.3	GQ2-F17	73.60	50.83	3.44	3.31
D20S164	20q13.32	GQ2-F18	101.00	57.05	2.56	3.42
D12S1300	12q23.1	GQ2-F17	104.00	98.50	3.39	3.23
D4S3360	4p16.3	GQ2-F17	0.00	0.12	3.35	2.83

^a^The genetic map position was based on Marshfield Medical Genetic marker set 11.

^b^Physical genomic position was retrieved from the UCSC database with human build GRCh37/hg19.

### Potential CAD-related genes underlying six significant CAD loci

To explore candidate genes for CAD under the six significant genetic loci identified for CAD in the combined GeneQuest II families, we annotated all genes underlying each linkage. Genetic intervals of the six linkages were converted to physical locations according to the genetic maps generated by the HapMap 2 project (lifted over to hg19). RefSeq genes located under the six linkages were retrieved from the UCSC database (Tack: RefSeq Genes; Assembly, GRCh37/hg19), and then evaluated for potential relationship with cardiovascular diseases using the online program DisGeNET^42, 43^. Counts of RefSeq genes and gene-disease pairs with score of >0.001 are summarized in Table 5.

**Table 5:** Summary of RefSeq Genes Related to Cardiovascaulre Diseases Under Each Linkage for CAD in the Combined GenQuest II Families.

CAD Locus (Genetic Map^a^)	Genomic Region^b^	RefSeq Genes^c^	Genes Related to Cardiovascular diseases^d^
**17q21**.**2** (56.9–83.1 cM)	34.40–57.50 Mb	514	*CCL3*, *CCL4*, *CCL3L3*, *CCL4L1*, *CCL4L2*, *CCL3L1*, *TADA2A*, *PLXDC1*, *TCAP*, *PNMT*, *PGAP3*, *ERBB2*, *IKZF3*, *CSF3*, *MED24*, *THRA*, *NR1D1*, *CCR7*, *KRT12*, *KRT20*, *GAST*, *HAP1*, *JUP*, *FKBP10*, *CNP*, *KCNH4*, *HCRT*, *STAT5B*, *STAT5A*, *STAT3*, *ATP6V0A1*, *MLX*, *RAMP2*, *WNK4*, *BECN1*, *AOC3*, *BRCA1*, *SOST*, *PYY*, *G6PC3*, *HDAC5*, *GRN*, *ITGA2B*, *FZD2*, *ADAM11*, *GJC1*, *CCDC103*, *GFAP*, *HEXIM1*, *MAP3K14*, *CRHR1*, *MAPT*, *WNT3*, *GOSR2*, *MYL4*, *ITGB3*, *MRPL10*, *PNPO*, *MIR10A*, *UBE2Z*, *GIP*, *IGF2BP1*, *B4GALNT2*, *ZNF652*, *NGFR*, *ITGA3*, *PDK2*, *SGCA*, *COL1A1*, *XYLT2*, *CACNA1G*, *LUC7L3*, *NME1*, *MMD*, *AKAP1*, *MPO*, *MIR142*
**7p22**.**2** (1.4 –13.0 cM)	0.88–7.25 Mb	87	*GPER1*, *MAFK*, *NUDT1*, *GNA12*, *SDK1*, *ACTB*, *AIMP2*, *EIF2AK1*, *RAC1*
**2q33**.**3** 191.9–202.4 cM	177.33–192.47 Mb	88	*NFE2L2*, *PDE11A*, *RBM45*, *TTN*, *CCDC141*, *ZNF385B*, *ITGA4*, *NEUROD1*, *PDE1A*, *FRZB*, *ITGAV*, *ZSWIM2*, *CALCRL*, *TFPI*, *COL3A1*, *COL5A2*, *SLC40A1*, *PMS1*, *MSTN*, *STAT1*, *STAT4*
**3q29** (206.5–216.0 cM)	188.96–193.86 Mb	31	*TP63*, *CLDN16*, *UTS2B*, *HRASLS*, *OPA1*
**5q13**.**2** (74.9–80.0 cM)	66.68–71.63 Mb	39	*PIK3R1*, *CCNB1*, *OCLN*, *SMN2*, *SMN1*, *NAIP*, *MCCC2*, *CARTPT*
**9q22**.**33**(103.6–105.3 cM)	101.72–104.22 Mb	20	*TGFBR1*, *NR4A3*, *INVS*

^a^Genetic map based on the Marshfield Medical Genetics database (http://research.marshfieldclinic.org/genetics/GeneticResearch/screeningsets.asp).

^b^Physical map based on hg19 by Adam Auton in 1000 Genome project (https://github.com/joepickrell/1000-genomes-genetic-maps).

^c^ReSeq genes retreived from the UCSC RefSeq database (Track: RefSeq Genes; assembly: GRCH37/hg19).

^d^Related genes were explored using program DisGeNet. Any gene-disease pair with a score of >0.001 was defined as a valid hit.

## Discussion

Identification of new genetic loci for CAD is critical for addressing the important issue of “missing heritability” in the field of genetics, and in fully elucidating the genetic basis of CAD. In this study, we report a unique genome-wide linkage scan for CAD in 24 large, multigenerational families from a well-characterized U.S cohort (GeneQuest II). We carried out a model-free NPL-all scan and identified six susceptibility loci for CAD on chromosomes 2q33.3, 3q29, 5q13.2, 7p22.2, 9q22.33 and 17q21.2. It is interesting to note that the 3q29 and 9q22.33 loci were previously identified by us in a genome-wide linkage scan for CAD in 428 nuclear families in the GeneQuest population^40^. Suggestive evidence of linkage to the 3q29 CAD locus (*P* = 2.0 × 10^−4^) was also found in a meta-analysis of four GWLS in Finnish, Mauritan, Germany, and Australian cohorts^44^. Therefore, the present study provides strong validation of the 3q29 and 9q22.33 linkages for CAD using an independent, large family-based linkage scan, suggesting that these two loci can be prioritized for identifying the underlying causative genes for CAD. Candidate genes for CAD at the 3q29 and 9q22.33 loci are listed in Table 5. There are 31 unique RefSeq genes annotated within the CAD locus on 3q29. DisGenNET analysis identified 5 genes related to cardiovascular diseases (Table 5). The *UTS2B* gene encodes Urotensin IIB and was shown to play a role in the acceleration of atherosclerosis development. Increased human Urotensin II levels were observed in hypertension, diabetes, atherosclerosis and CAD^45^. There are 20 unique genes within the 9q22.33 locus and three genes (*TGFBR1*, *NR4A3*, *and INVS*) were linked to cardiovascular diseases (Table 5). *TGFBR1* encodes transforming growth factor beta receptor 1 (TGFβ1) and an increase in active TGFβ1 levels were correlated with both the occurrence and severity of CAD^46^.

The four other CAD loci on 2q33.3, 5q13.2, 7p22.2 and 17q21.2 are all novel. The chromosome 17q21.2 linkage is the most significant locus for CAD identified in this study. The 17q21.2 CAD locus was initially identified at marker D17S1299 at the position of 62.01 cM with a two-point NPL score of 6.20 and a multipoint NPL score of 5.38 (Table 2). This CAD locus spans a large genetic interval of 26.2 cM (corresponding to 34.40–57.50 Mb) (Fig. 3). Within the 17q21.2 CAD locus, we found SNP rs46522, which is a CAD-risk variant identified by a large-scale GWAS for CAD in 2013^12^ and located about 8 Mb away from D17S1299. SNP rs46522, located in the *UBE2Z*-*GIP*-*ADTP5G* gene cluster, exhibited a strong cis-eQTL (expression quantitative trait locus) to *UBE2Z* in whole blood samples and to *ATP5G1* in left ventricle samples according to the GTEx database v6^47^. On the other hand, we identified a set of 514 unique RefSeq genes within the 17q21.2 CAD locus; 77 of them were linked to cardiovascular diseases based on data from DisGenNET (Table 5). In particular, *CCL3* and *CCL4* encoding small CC chemokines known as macrophage inflammatory protein 1α and 1β, respectively, were well-recognized as key mediators of both diabetes and atherosclerotic cardiovascular disease^48^. Elevated expression levels of both *CCL3* and *CCL4* were found in atherosclerotic lesions in *ApoE* ^−/−^ mice^49^. Leukocyte-derived CCL3 can induce neutrophil chemotaxis toward the atherosclerotic plaque, causing accelerated lesion formation^50^. *CCL4* was also upregulated in atherosclerotic plaques in stroke patients^51^. *NR1D1* is also a candidate gene for CAD. It is located 600 kb from marker D17S1299, encodes a member of the nuclear receptor superfamily and regulates genes involved in triglyceride metabolism, inflammatory and the pathogenesis of atherosclerosis^52^. NR1D1 can regulate apolipoprotein APOC3 via binding to the proximal promoter^53^. Future studies may focus on these strong candidate genes to identify causative genes that contribute to the risk of CAD in families.

The second most significant linkage for CAD on 7p22.2 was identified with marker D7S3056 at a position of 7.44 cM (physical position: 4.49 Mb) with two-point NPL score of 5.19 and a multipoint NPL score of 4.74 (Table 2). This is a novel locus for CAD. No GWAS variants were found to be located within the 7p22.2 locus. The closest GWAS SNP for CAD was rs2023938 in *HDAC9*, which is located at 7p21.1^13^. There are 87 unique RefSeq genes located within the 7p22.2 locus (0.88 Mb to 7.22 Mb). DisGenNET analysis identified 9 genes related to cardiovascular diseases (Table 5). *SDK1* was found to be associated with hypertension in the Japanese population^54^. *GPER1* encodes a multi-pass membrane protein that is localized to the endoplasmic reticulum and *Gper1* knockout mice showed increased atherosclerosis progress and vascular inflammation^55, 56^.

The 2q33.3 locus, represented by marker D2S1384 at 200.43 cM (physical position: 205.23 Mb), covers a genomic region of 15.14 Mb (Table 2 and Fig. 2). GWAS found that SNP rs6725887 in *WDR12*, which is only 1.48 Mb from marker D2S1384, was associated with early-onset MI and ischemic stroke^7, 12, 57^ at a genome-wide signifcance level. Moreover, DisGenNET analysis identified 21 genes related to cardiovascular disease (Table 3). *PDE1A* encodes a cyclic nucleotide phosphodiesterase and differential expression of *PDE1A* was observed in human epicardial adipose tissues from male patients affected with CAD^58^. *TFPI* encodes a tissue factor (TF)-dependent pathway of blood coagulation^59^. An elevated plasma TFPI level was significantly associated with the presence and severity of CAD^60, 61^. *TFPI* expression can be regulated by *ADTRP*, a CAD susceptibility gene identified by our group^17^.

The 5q13.2 locus was mapped at marker GATA138B05 at 78.80 cM (or 71.40 Mb) and spanned an interval of 5.1 cM (4.95 Mb) (Fig. 2). This is a novel locus for CAD. DisGenNET analysis identified 8 genes linked to cardiovascular diseases at the 5q13.2 locus (Table 5). *PIK3R1* encodes Phosphoinositide-3-Kinase Regulatory Subunit 1 and was predicted to be a cardiovascular disease-related gene by a network topology analysis^62^. *PIK3R1*is a target of miR-221, and a recent small RNA sequencing analysis revealed that the *miR*-*221*- *PIK3R1*pair was deregulated in late endothelial progenitor cells (late EPCs) of CAD patients^63^. *CCNB1* encodes a regulatory protein involved in mitosis and a recent study showed that genetic variants in *CCNB1* contributed to risk of the restenosis of intracoronary stents^64^.

The compelling results above demonstrated that linkage analysis with fewer but larger pedigrees can achieve comparable performance with hundreds of small nuclear families. As shown in Table 2, the 3q29 and 9q22.33 CAD loci were identified by both GWLS with 24 large families (GeneQuest II) and by a similar analysis with 428 nuclear families in the GeneQuest population^40^. Our results also demonstrate that GWLA has a comparable power to GWAS. The 2q33.3 and 17q21.2 CAD loci, which were identified by the GWLS with 24 large families here (GeneQuest II) and represented by D2S1384 and D17S1299, respectively, contain CAD-risk SNPs identified by GWAS (rs6725887 at 2q33.3 and rs46522 at 17q21.2) (Table 2, Figs 2 and 3). Therefore, we conclude that increasing family members within individual families can markedly improve the power for identifying disease linkage and loci. These data also suggest that our GeneQuest II database is a promising resource for identifying novel risk genes for CAD. Future studies on fine mapping and targeted sequencing will uncover causative variants or genes for CAD at the CAD loci identified in this study.

We also carried out genome-wide linkage analysis in each GeneQuest II family and found that each of the six significant CAD loci identified in the combined family cohort (Table 1) were also identified in at least one individual family (Table 3). For example, the top two CAD loci on chromosomes 17q21.2 and 2p22.2 were observed in two families (the best NPL score = 16.81) and three individual families (the best NPL score = 12.42), respectively. Moreover, individual family-based analyses also identified 15 new, significant linkages in 5 families that were not captured by joint linkage analyses of 24 GeneQuest II families, including 7 highly significant linkages, 2 significant linkages, and 6 suggestive significant linkages (Table 4). None of the 15 new genetic loci have been previously reported for CAD. Of interest, the two top ranked CAD locus on 12q13.13, represented by D12S297 (multipoint NPL score = 6.76) and 17q22 represented by D17S1290 (multipoint NPL score = 6.51), were linked to CAD-associated traits of body mass index (BMI)^65^ and metabolic factors^66^.

Despite a list of significant CAD loci identified in this study, there were several limitations. First, the density of microsatellite markers in this study was low (10 cM per marker). Future fine mapping studies may be carried out with additional markers surrounding the microsatellite polymorphisms used for linkage analysis or SNP microarrays with a much increased marker density. Single SNPs may not as informative as microsatellite markers for linkage analysis due to their bi-allelic status, but haplotypes constructed using multiple SNPs may be considered as multi-allelic markers^67^. Fine mapping will confirm that the linkage loci are overlapping in different families, shorten and narrow the linked regions (if shared) and eventually reduce the number of candidate genes for some loci. Moreover, fine mapping with SNP arrays may allow us to compare the SNP linkage data with the top hits from previous GWAS and identify new SNPs associated with CAD. Similarly, ongoing whole genome sequencing may be another powerful approach to capture SNPs or causal variants associated with CAD in the 24 GeneQuest II families. Second, we highlighted 3–77 genes at each CAD locus based on the evidence from existing literature with a purpose to illustrate the relevance of each CAD locus to etiological process of CAD. However, the CAD causal genes being responsible for each linkage were possibly overlooked in this study (Table 5). Third, the 24 GeneQuest II families were of European descent, and it is likely that some significant CAD loci may not be expanded to other ethnic populations.

In summary, we report the results of a genome-wide linkage scan of 24 large GeneQuest II families and uncover six genetic loci for CAD on chromosomes 2q33.3, 3q29, 5q13.2, 7p22.2, 9q22.33 and 17q21.2. Our study identifies four novel CAD loci (2q33.3, 5q13.2, 7p22.2 and 17q21.2). Similar analysis in individual families confirmed the six significant CAD loci and also identified nine new significant linkages on 2q11.2, 9p24.2, 9q34.2, 11q14.1, 12q13.13, 15q26.1, 17q22, 20p12.3, and 22q12.1. Our study also independently confirms the 3q29 and 9q22.33 CAD loci identified by our earlier genome-wide linkage scan for CAD in 428 nuclear families. Two loci on 2q33.3 and 17q21.2 contain GWAS risk variants identified from population samples. These studies may provide a new framework for uncovering causative variants, genes and biological pathways involved in the pathogenesis of CAD.

## Methods

### Study participants

Twenty-four large, extended, and multigenerational CAD families were recruited at the Center for Cardiovascular Genetics of the Cleveland Clinic. The study was referred to as GeneQuset II to distinguish it from the original GeneQuest study which recruited more than 428 nuclear families, mostly for sib-pair analysis. The GeneQuest II study started in the year of 2001 and is completely independent from the earlier GeneQuest study carried out between 1995 and 2000. This study was reviewed and approved by the Cleveland Clinic Institutional Review Board (IRB) on Human Subject Research, and conformed to the guidelines set forth by the Declaration of Helsinki. Written informed consent was obtained from all participants.

Clinical phenotypic evaluation of study participants was carefully carried out by a panel of cardiologists. The presence or absence of CAD was assessed according to coronary angiography with >70% stenosis, a history of revascularization procedures such as percutaneous coronary angioplasty (PCA) or coronary artery bypass (CABG), and a previous diagnosis of myocardial infarction (MI) as described^35, 68, 69^. Families or patients with hypercholesterolemia, insulin-dependent diabetes, childhood hypertension, and congenital heart disease were excluded from this study. Each family has at least four definitely diagnosed CAD patients; and the average pedigree size was 18. Clinical and demographic features of the 24 GeneQuest II CAD families with 433 family members are summarized in Table 1. All recruited family members were Caucasians. The distinguishing features for the GeneQuest II cohort are large families with three or more generations, 100% whites and a well-balanced male versus female ratio (209/224). A total of 398 sibling pairs were generated in this cohort, including 154 sister/sister pairs, 105 brother/brother pairs and 139 brother/sister pairs. In contrast to sib-pair analysis of 428 nuclear families in our previous study^40^, genome-wide linkage analysis was carried out using all family members instead of sibling pairs only, given the large pedigrees collected in GeneQuest II (Fig. 1).

### Extraction of human genomic DNA and genotyping

Whole blood samples were drawn from each study participant. Genomic DNA was isolated using the Gentra Puregene blood (QIAGEN, Valencia, CA, USA). All DNA samples were quantified using NanoDrop 2000 (Thermo Scientific, Wilmington, DE, USA) and inspected for quality by agarose gel electrophoresis.

Genome-wide genotyping was performed by Mammalian Genotyping Service of the National Heart, Lung, and Blood Institute directed by Dr. James L. Weber at Center for Medical Genetics at Marshfield Clinic (http://research.marshfieldclinic.org/genetics/GeneticResearch/screeningsets.asp) using Screening Set 11. The screening set consists of 410 microsatellite markers spanning the whole human genome by every 10 cM on average.

### Linkage analysis

Prior to linkage analysis, raw genotyping data were cleaned as described in our previous studies^35, 40^. In brief, genotypes with non-consensus calls were re-genotyped or deleted. Microsatellite markers on sex chromosomes were excluded. Missing parental genotypes were added and treated as missing values to complete family pedigrees (Fig. 1) for linkage analysis. Mendelian inconsistencies were detected by using MARKERINFO built in software S.A.G.E (Statistical Analysis for Genetic Epidemiology)^70^. Genotypes with Mendelian errors were excluded from further genome-wide linkage analysis by Genehunter version 2.1_r2 beta^71^. Relationship between family members (i.e., sibling pairs, parents-offerings trios) within each family was verified by the RELTEST program included in the S.A.G.E software page^70^. The RELTEST program did not detect any inconsistent family relationship. Allele frequencies for all microsatellite markers were estimated by module FREQ in S.A.G.E in the pooled samples containing all of our existing family studies. Program Mega2^72^ was used to generate the input format required for Genehunter version 2.1_r2 beta^71^. Affected and unaffected individuals were coded as “2” and “1”, respectively, whereas individuals with uncertain phenotype were coded as “0”.

The principle of the Genehunter linkage analysis is to examine any excess of identity-by-decent^73^ allele-sharing between all affected subjects within a family. We used the NPL-all statistic within Genehunter version 2.1_r2 beta for linkage analysis, which examines all individuals in the 24 GeneQuest II families simultaneously and provides a more powerful test (www.broad.mit.edu/ftp/distribution/software/genehunter/). Without specifying the disease transmission model for all markers, non-parametric linkage (NPL) analysis was carried out to jointly analyze genotype data of all 24 GeneQuest II families. The linkage between CAD and a genetic marker was evaluated by calculating NPL score Z, which is the summation of standardized identity-by-descent allele-sharing scores across multiple families. Under a null hypothesis of no linkage, Z has mean 0 and variance 1 by choosing appropriate weighting factors. Statistical significance of Z can be inferred by comparing the observed Z against to its null distribution. Two types of NPL scores were calculated for each marker: 1) A two-point NPL score examined whether a single marker was linked to CAD; 2) A multipoint NPL score investigated whether a group of markers were linked to CAD. The advantage of the multipoint approach is its capability of incorporating the information of adjacent markers into linkage analysis (making markers more informative). The NPL-all linkage analysis was also carried out individually in each of the 24 GeneQuest II families. The larger a NPL score is, the stronger the linkage it indicates. As suggested by Lander and Kruglyak^74^, linkage peaks were defined in three categories: (1) Highly significant linkage: NPL of 4.99 (or *P* value of 3 × 10^−7^); (2) Significant linkage: NPL of 4.08 (or *P* value of 2.2 × 10^−5^); (3) Suggestive Linkage: NPL of 3.18 (or *P* value of 7.4 × 10^−4^).

### Public resources

UCSC database: http://genome.ucsc.edu/.

DisGenNET^42^: http://www.disgenet.org/web/DisGeNET/menu/home.

GTEx^47^ portal: http://gtexportal.org/home/.

Genetic map of microsatellite markers: http://research.marshfieldclinic.org/genetics/GeneticResearch/screeningsets.asp.

Physical map based on hg19: https://github.com/joepickrell/1000-genomes-genetic-maps.

Genehunter version 2.1_r2 beta: www.broad.mit.edu/ftp/distribution/software/genehunter/.
